# Development and psychometric properties of Iranian Women’s Quality of Life Instrument (IWQOLI): mixed exploratory study

**DOI:** 10.1186/s12889-023-17028-1

**Published:** 2023-10-25

**Authors:** FatemehSadat SeyedNematollah Roshan, Fatemeh Alhani, Armin Zareiyan, Anoshirvan Kazemnejad

**Affiliations:** 1 Nursing Department, Faculty of Medical Sciences, Tarbiat Modares University, Tehran, Iran; 2 Department of Nursing, Tehran Medical Sciences Branch, Islamic Azad University, Tehran, Iran; 3 Department of Community Health of Nursing School of, AJA University of Medical Sciences, Tehran, Iran; 4 Department of Biostatistics, Faculty of Medical Sciences, Tarbiat Modares University, Tehran, Iran

**Keywords:** Design, Quality of life, Questionnaire, Women

## Abstract

**Background:**

To determine the health needs and promote women's health, their quality of life should be investigated. For this purpose, a valid tool is needed, that has credible validity and reliability, and its concepts are clearly defined and culturally appropriate. This study aimed to develop and assess the psychometric properties of “Iranian Women’s Quality of Life Instrument (IWQOLI)”.

**Methods:**

The items of “IWQOLI” were generated from themes extracted (150 items) from a content analysis approach with the participation of 40 women. Face validity of the questionnaire with the participation of 10 women and content validity by 10 experts was approved. To determine the domains of the questionnaire, exploratory factor analysis (principal component extraction method) was performed. Internal consistency and test—retest reliability methods with 14-day intervals (30 women) were used to assess the reliability of WQOLI.

**Results:**

After performing the face and content validity, 32 items were deleted. S-CVI/Ave was obtained for the instrument (0.93). The factor structure of the inventory was identified by undertaking a principal component analysis in a sample of 590 women. Five factors were extracted with a total variance account of 56.24% and 28 items dropped at this point. The IWQOLI score was significantly correlated with the SF-36 (*r* = 0.717, *p* < 0.001). Reliability was demonstrated with Cronbach’s alpha coefficient of 0.919 for the entire scale (90-item). Consistency of the instrument was established with test–retest reliability with an interval of 2 weeks (intra-cluster correlation = 0.889, *P* < 0.001).

**Conclusions:**

The Iranian women’s Quality of life Instrument “IWQOLI”, consisting of 90 items representing 5 domains (sense of peace in life, sense of security, health responsibility, pleasant communication, received comprehensive support), demonstrated excellent psychometric properties, so it may be used for measuring women’s QOL in practical research.

## Introduction

In recent years, the healthcare system, which is responsible for the health and treatment of all populations, has focused on preliminary health and quality of life (QOL) [1]. Since the quality of life, health and disease mutually affect each other, by examining the quality of life, appropriate and related interventions can be made to prevent many health problems and maintain and improve the health of people [2]. But the quality of life varies from a person's point of view in different situations, and it relates to the individual's satisfaction from his or her life and is related to factors such as age, culture, gender, education, class status, social environment, and health and illness. Among the factors mentioned, the gender issue plays a key role in determining life quality. According to the World Health Organization, women are considered one of the high-risk groups in the community because of their many roles in the family and society, going through various physiological crises such as puberty, menstruation, pregnancy, labor, and menopause and also a greater risk of suffering from poverty, hunger, and malnutrition, heavy workload and gender discrimination [3]. In addition, the participation of women in social and political affairs has also increased significantly. These all situations could have important effects not only on the quality of life but also on the health status of women [4, 5].

Several studies have been conducted in this regard in the population of women as a high-risk group, which in general confirms the fact that there are diversities in the quality of life between men and females. For example, the results of studies in the general population indicate a weaker quality of life for women in all dimensions compared with men. The possible causes are stated as the limited physical activity of women outside the home, the greater sensitivity of women in dealing with adverse events, and other sociocultural factors related to gender [6, 7].

Since changing the quality of life of women as a subjective understanding of the life situation can have wide consequences not only on their health but also on the whole of society, it should be given special attention [8]. According to the literature review, to measure women's quality of life, researchers have used general quality of life instruments or general health questionnaires like SF_36_, WHOQOL-BREF [9, 10], and the Quality of Life Scale(QOLS) [11] that can be used for both men and females. However, measuring the quality of life with public tools does not provide specific information based on the specific gender characteristics of this society.

In the meantime, other specific tools have been designed for the quality of life of women suffering from various health complications, such as the fertility quality of life (FertiQoL) tool [12] and a questionnaire to assess the female quality of sexual life [13] or Maternal Postpartum Quality of Life [14] and also Menopause-Specific Quality of Life (MENQOL) Questionnaire [15], and instrument for assessing QOL of women with urinary incontinence(I-QOL) [16]. But none of these tools examines the status of women's quality of life before getting sick at the community level, in other words, at the level of preliminary prevention. Paying attention to the economy of health by reducing treatment costs is one of the criteria for the development of any society, and the way to achieve it is to pay attention to health at the level of preliminary prevention [17].

The point is that, according to the reviewed studies, none of the general quality-of-life tools are designed based on the lived experience of women, and on the other hand, there is no specific tool based on the culture of Iranian women based on their special needs, which may be the reason for the lack of clarity in the definition of the women's quality of life in Iran. Therefore, this research is aimed at determining the nature of Iranian women's quality of life by conducting an in-depth qualitative analysis of their experiences and then designing and psychometrically analyzing a native instrument based on Iranian women's culture.

## Methods

### Design

**Fig. 1:**
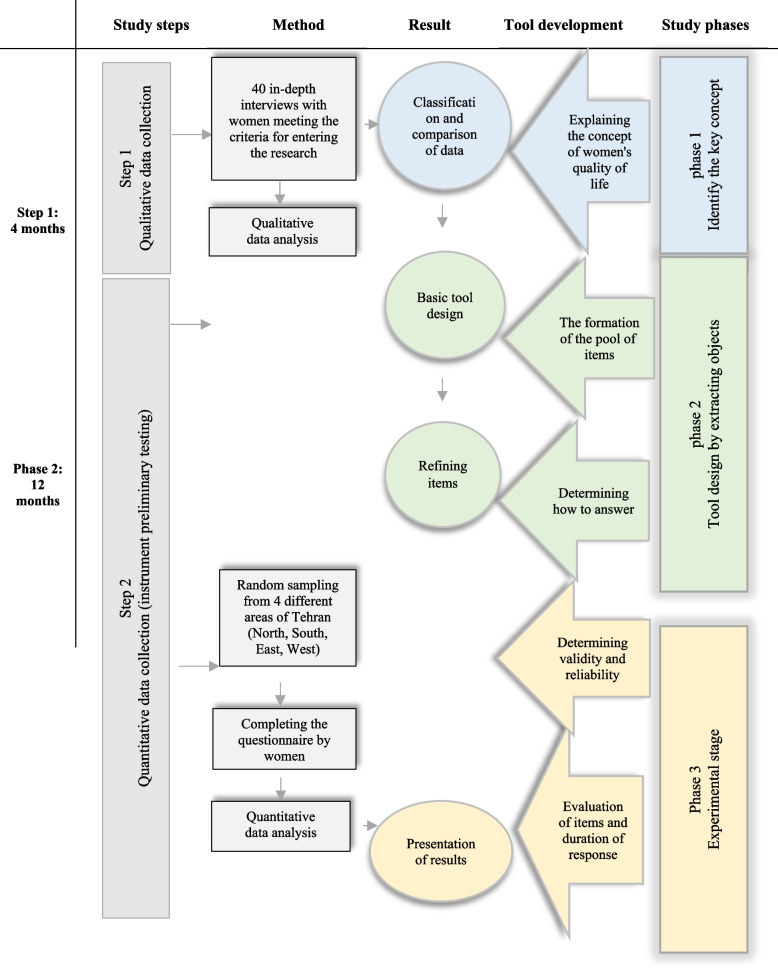
A summary of steps to develop and assess psychometric properties of IWQOLI

This is a mixed study that was conducted by the Creswell method in two steps with three phases (1- qualitative phase by content analysis, 2- development of item pools, 3- quantitative phase to determine the psychometric properties of the tool) [18] from May 2016 to January 2018. A summary of steps to develop and assess the psychometric properties of IWQOLI is illustrated in Fig. 1.

In the first phase, to identify the key concept (women’s QOL), we used conventional qualitative content analysis. Participants in the study were selected from neighborhood halls, sports halls, cultural centers, mosques, parks, sports halls, and educational places such as universities, schools, and educational institutions in the city of Tehran, Iran, through purposive sampling.

Inclusion criteria in the study were all women aged 15 to 60 years with the ability to read and speak Persian, without any disabilities and other physical and psychological ailments, excellent ability of communication and expression, and willingness to do an interview (45 to 60 min) and talking about their life situation, and their worries and concerns in their daily life (totally 40 women when we reach data saturation). To clarify, based on the entry criteria, the participants were gradually entered into the study, and individual and face-to-face interviews were conducted until no new code was obtained and data saturation was done with 38 participants. In the last 2 interviews (interviews 39 and 40), the researcher found that the data did not provide him with new information.

With the consent of the participants, the interviews were recorded using an mp3 player and to better encompass the feelings and experiences of the participants (immersion in data), they were heard several times within 24 h after the interview, then were typed using WORD computer software and reviewed several times. To clarify and fill the gaps between the findings, another meeting was arranged by asking follow-up questions (about 20 min), and the necessary data was completed. After collecting the data, it was transferred to the MAXQDA software version 10.

The interview was conducted in the following order. First, to warm up the atmosphere of the interview and gain more confidence from the participants, a pre-interview was conducted in the form of greetings and normal daily conversations, then demographic questions were asked with the variables of age, marital status, education, occupation and place of residence, etc.

In the second stage of the interview, basic questions related to the research were asked as follows:

- Please tell me about your life situation.
- Has something happened in your life that has changed your normal life?

After analyzing the data, the questions were focused on the obtained concepts.

- What communication methods do you use in your daily life?
- What does support mean in your quality of life?
- What does health responsibility mean in your life?
- Do you feel safe in your life?

And other questions were asked in the same fields. Then, to clarify the answers of the participants, probing and follow-up questions were also asked.

- Please explain more, what did you mean?
- Give an example so that I can better understand what you mean.

After the completion of the interview, when the researcher was sure that there was no specific data, entered the post-interview phase and the participant was asked:

- was there a specific question that I did not ask?
- Is there something special that you prefer to express?

At this stage, after collecting information through interviews, Zhang's systematic and transparent method in 8 steps (Preparing the data, Defining the Unit of Analysis, developing categories and a Coding Scheme, testing your Coding Scheme on a Sample of Text, Code all the Text, Assess Coding Consistency, Draw Conclusions from the Coded Data, Report Methods and Findings) was used to process the data [19].

### Rigor

The important issue in examining qualitative research is how much the reported results are a reflection of reality. Guba and Lincoln (1985) mention four indicators of credibility, confirmability, transferability, and dependability as a subset of the accuracy of findings. which was also examined in this study [20]. In the first phase of the research, the credibility of the collected data was confirmed through long-term engagement with the data, in-depth data analysis, combining information sources, and using multiple data collection methods such as interviews, colleague review and to ensure the transferability of the findings tried to select participants in divers areas and also asked two women with similar criteria to review and confirm the obtained information. To ensure the confirmation of the findings for others the study process was recorded and reported carefully. To verify the dependability, all the data were analyzed continuously step by step and controlled by two external observers [21].

In the second phase, from all items that were obtained inductively through interviews and terms of the participants, the main themes and pool of items were formed. Then, with the research team's opinion, the conceptually similar concepts were removed or merged. In this way, the initial tool for evaluating women's quality of life was designed based on perceived definitions in the form of a methodological process.

In the third phase, the psychometric characteristics of the tool were examined based on the COSMIN criteria, including validity (face, content, construct, criterion), reliability (internal consistency, test–retest), responsiveness (time, intrusiveness, cost, method of administration and the ability to respond).

#### Face validity

Qualitative face validity was determined by asking the points of view of 10 women in the target group. Furthermore, a quantitative assessment of face validity was conducted with the calculation of impact scores (Impact score = frequency(%)× importance) to reduce and remove inappropriate items. The question that received a score of above 1.5, was identified as an important item [22].

#### Content validity

In terms of qualitative content validity, 10 experts in the field of health, quality of life, nursing, and questionnaire designers were asked to write their editorial opinions in writing after carefully studying the instrument (about grammar, Wording, Scaling, and Item Allocation). To assess quantitative content analysis, the content validity ratio (CVR) was used as well as the content validity index (CVI). To determine CVR, 10 experts were asked to score each item based on the three-part spectrum (essential, useful but not necessary, and not necessary). CVR was calculated through the bellow formula.

$$CVR=\frac{n_E-\frac{N}{2}}{\frac{N}{2}}$$

According to the Lawshe table, the minimum value was determined as 0/62 [23].

Then based on Waltz and Bausell's criteria, CVI (Item content validity index & and scale content validity index were calculated. To assess I -CVI, 10 experts scored the relevancy, of each statement in WQOLI using a 4-point rating scale (1 = not relevant, 2 = somewhat relevant, 3 = quite relevant, and 4 = extremely relevant), Then, the score for each item was calculated by dividing the number of specialists agreeing with the phrase having grades 3 and 4 on the total number of specialists. A rate of 0/78 and above is appropriate [22, 24]. CVI was calculated according to the bellow formula:

$$CVI=\frac{number\,of\,raters\,giving\,a\,rating\,of\,3\,or\,4}{total\,number\,of\,raters}$$

The Scale Content Validity Index Average method (S-CVI/Ave), was used to determine the average value of I-CVI for the whole scale. A reference value of more than 0.90 is desirable [22]. Based on the results of the pilot study and experts’ opinions, necessary changes were made the WQOLI was modified accordingly and the final instrument was obtained.

#### Construct validity

In this research, the exploratory factor analysis (EFA) method was used to discover and identify the indicators as well as the relationships between them. The number of respondents required for factor analysis was 5–10 for each statement [25]. At this stage, a random sample consisting of 649 women (5.5 times the initial subjects including a 10% attrition rate) was considered. 590 of them completed the questionnaire completely (with a response rate of 91%) from November 2017 to January 2018.

For indicating the degree of susceptibility of data for factorial analysis and sampling adequacy, the Kaiser–Meyer–Olkin (KMO) and Bartlett Sphericity tests were used. The recommended KMO value was 0.6. A Scree plot was used to predict the number of factors. To determine the number of questionnaire constructs eigenvalues greater than 1.0 were considered by the principal component extraction method. The varimax method was used for orthogonal rotation of the acquired constructs [22]. For all 118 items factor-loading > 0.4 was considered.

#### Criterion validity

In the validity of the criterion, the relationship between the current instrument with the other criterion is tested [22]. In this study, a standard instrument (SF_36_) was used to measure criterion-dependent validity. For this purpose, every 590 participants in this study were asked to complete the WQOLI in addition to SF_36_ as a completion criterion. Then, using the Pearson correlation test, the scores of the two instruments were compared.

#### Reliability

We used internal consistency and test—retest reliability methods with 14-day intervals to assess the reliability of WQOLI. Internal consistency was evaluated using Cronbach's alpha coefficient (0.70 or more) [26]. In this research, Cronbach’s alpha of WQOLI was calculated in a sample of 30 women (5% of the samples) using a convenience sampling method. It was measured for each factor and also for the whole of the questionnaire. Values higher than 0.70 were considered desirable.

The stability of a tool shows the reliability of obtained scores following a test–retest administration. In this step, the stability of WQOLI was assessed using the test–retest reliability measurement method. 30 women filled out the questionnaire on two different occasions, at 14-day intervals, and the Persian correlation coefficient was calculated. Values higher than 0.70 were considered desirable [22]. Finally, the collected data were imported into version 22.0 for further analysis.

#### Responsiveness

The response to the questionnaire was examined through the criteria of time, intrusiveness, cost, method of administration, and the ability to respond. Time means the time that people need to complete the questionnaire. A long period leads to the fatigue of the respondents and answering the items without paying attention. The intrusiveness of the questionnaire is important in terms of how well the items fit the culture of the target group. In some conservative cultures, people sometimes feel some items are a threat to them, do not answer, or even withdraw from participating in the study. The cost is important in terms of copying the questionnaire or the cost of using an assistant to collect information. The method of use is important in terms of whether the tool is designed in such a way that the participant can answer the questions easily. Or if the respondent is unable to answer the questionnaire due to being illiterate or having a physical disability such as blindness, etc., can another person read the questions to the main participant? [27, 28]. In the current research, during the validity and reliability process of the questionnaire, the number of missing and unanswered items by the participants and the time required to complete the questionnaire were calculated.

## Results

In the qualitative stage, analysis of the content of the data from interviews with 40 women led to the explanation of the concept of women's quality of life. Accordingly, the quality of life of women is “a dynamic conception of mental perception of lives, that expressing her experience of having a health responsibility and pleasant communicating (with themselves, God, the environment, and others), combined with an experience of a sense of security, that has been received in the context of comprehensive support which is in fluctuated in different periods of life”.

In the quantitative phase, out of 649 women who signed the informed consent letter; 590 individuals completed and returned the questionnaire packet, and 59 people did not complete it (response rate 91%).

The average age of the 590 study participants was 35.08 ± 9.17 (± SD) years (range: 15–49 years). Regarding marital status, most of them were married (53/4%), 39.8% were single, 6.4% were divorced and only 2 women were widowed. Regarding the 6 levels of education (below high school, high school graduate, two-year college, bachelor, master, and Ph.D.), most of the women had bachelor's degrees (38.5%) and the minority had below high school degrees (4.6%). Regarding employment status,41% were housewives, 32.4% had part-time and 26.6% had full-time jobs. 47.1% of women had an average monthly income of 10 million rials. 67.3% lived in rented houses and the rest were landlords or lived in government houses.

After the interview, 450 items were extracted from the qualitative data, then the extracted items were assessed by the research team in three sessions, and phrases with overlapping concepts were merged. Therefore, the terms of the original tool were reduced to 150 phrases.

### Validity

In qualitative face validity, appropriate comments made by women were subject to necessary changes. Subsequently, the quantitative face validity 6 items were eliminated due to the impact score of less than 1.5. In the qualitative content validity, the items were corrected according to the opinion of the specialists.

In quantitative content validity, by determining the CVR, 26 items were eliminated due to score points less than 0.62. Then, CVI was calculated. At this stage, all questions obtained high scores (0.9) and there was no eliminated question. It should be noted that the S-CVI/Ave was 0.93. Eventually, at the end of the determination of face and content validity, the items were reduced to 118, in four areas “received comprehensive support (16 items), pleasant communication (42 items), sense of security (23 items), and health responsibility (37 items).

### Factor analysis

**Fig. 2:**
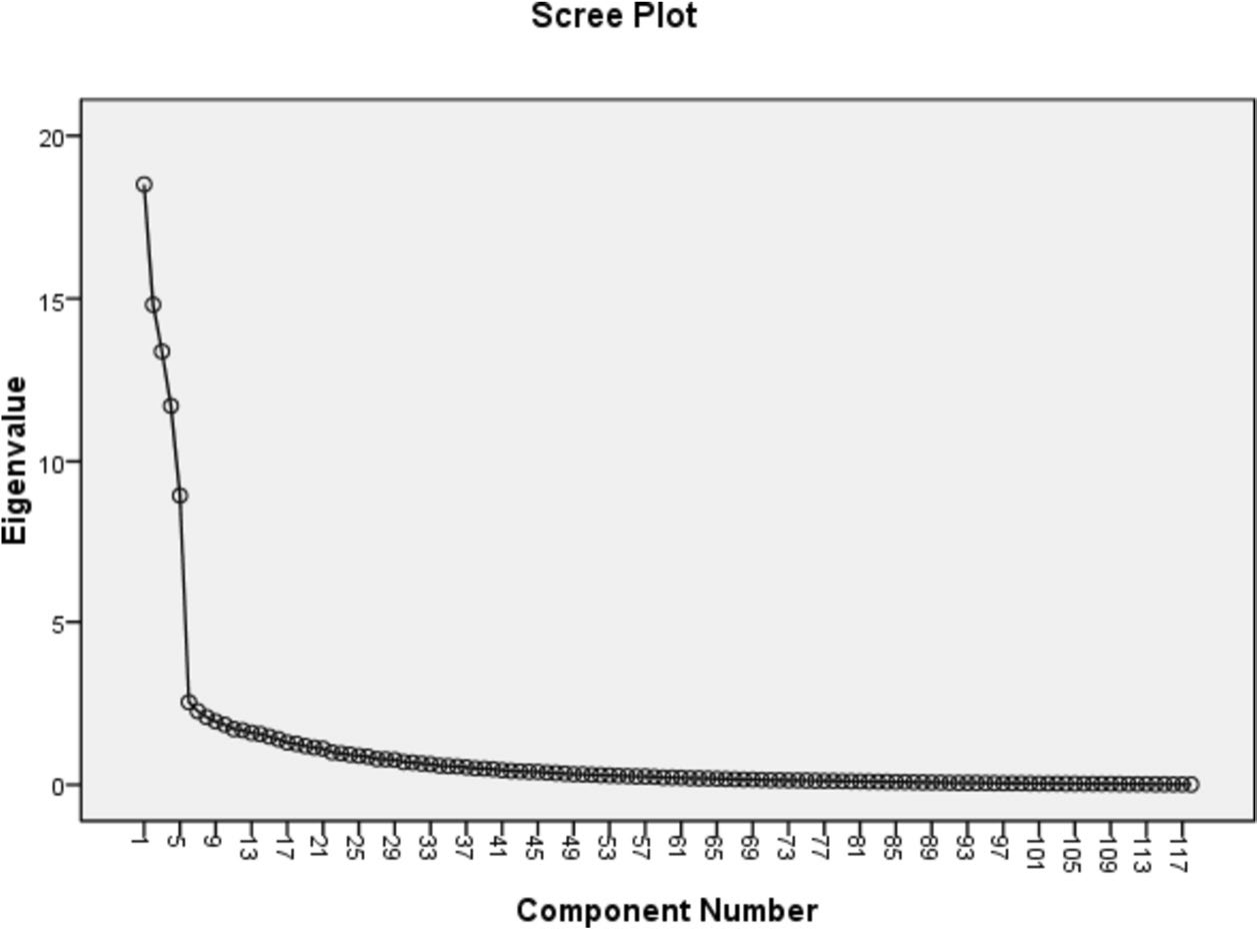
Scree plot of the exploratory factor analysis of IWQOLI

The KMO value by Exploratory Factor Analysis was 0.904; this exceeding the recommended value of 0.6 indicated the sampling adequacy. Five components with Eigen values exceeding 1, were indicated by principal components analysis. The scree plot illustrated in Fig. 2 also suggested 5 factors that became the default for factor analysis. Orthogonal rotation was used through the Varimax procedure, to facilitate the interpretation of these five components (Table 1). The five factors explained 56.25% of the variance. These five factors are labeled “sense of peace in life, sense of security, health responsibility, pleasant communication, and received comprehensive support” (Table 2).

**Table 1:** Total variance explained for five factors

Component	Initial Eigenvalues			Extraction Sums of Squared Loadings			Rotation Sums of Squared Loadings		
	Total	% of Variance	Cumulative %	Total	% of Variance	Cumulative %	Total	% of Variance	Cumulative %
1	18.507	15.684	15.684	18.507	15.684	15.684	17.647	14.955	14.955
2	14.813	12.553	28.237	14.813	12.553	28.237	14.872	12.603	27.558
3	13.374	11.334	39.571	13.374	11.334	39.571	12.542	10.628	38.187
4	11.705	9.919	49.491	11.705	9.919	49.491	12.426	10.531	48.718
5	8.947	7.582	57.073	8.947	7.582	57.073	8.884	7.529	56.247

**Table 2:** Extracted factors/factor analysis using varimax rotation (samples)

number	Load factor	Items of the first factor: sense of peace in life
Q50	.906	I feel relaxed with prayer
Q9	.854	I am satisfied with the power of my concentration and learning
Q1	.845	Because physical pain does not prevent me from doing my daily routine, I feel so good
Q5	.811	I am pleased to be able to keep my peace in stressful situations
Q3	.787	The more people's attitude is that I have a balanced body
Q11	.536	I am optimistic and I have a positive view of everything
		Items of the second factor: sense of security
Q84	.928	I am pleased when my opinions are taken into consideration at work
Q98	.892	I feel calm about being in the safe neighborhood
Q87	.804	I'm pleased to be successful in my work
Q99	.798	Going home makes me relax
Q97	.787	I am pleased to have no concern for the provision of housing
Q96	.772	I am pleased to receive a fair remuneration
		Items of the third factor: health responsibility
Q16	.921	I am pleased to be able to address my favorite hobbies
Q15	.911	I feel relaxed about having a comfortable sleep
Q21	.890	I am glad to have enough time to exercise
Q29	.880	Benefiting from religiously meals is important to me
Q28	.803	I feel well that the food I eat is safe and healthy
Q33	.794	Using medication prescribed by a doctor is more reliable for me
		Items of the fourth factor: pleasant communication
Q70	.836	I established a friendly relationship with others easily
Q75	.817	I am satisfied with my marital status
Q57	.805	Presence in areas with flowers and plants make me feel good
Q56	.802	Dealing with flowers and plants makes me calm down
Q65	.779	I am pleased that others give me opportunity for talking
Q58	.630	Living in a pleasant climate is pleasant to me
		Items of the fifth factor: received comprehensive support
Q105	.820	I feel calm, because of government assistance to cover my living expenses,
Q112	.810	I am pleased to be able to share my problems with family
Q104	.790	I am pleased that my wife and relatives give me the things I need
Q117	.789	Relative assistance in my everyday life made me happy
Q116	.785	I have access to the news and information necessary for my daily life
Q103	.755	I am pleased to be financially secured

### Reliability

After the exclusion of 28 items, the score of WQOLI was remarkably correlated with the SF-36 (*r* = 0.717, *p* < 0.001) (Table 3). The Cronbach’s alpha was 0.919 which showed the high reliability and strong internal consistency of this scale (Table 4). The test-re-test reliability resulted in a coefficient of (*r* = 0.889; *P* < 0.001) (Table 5).

**Table 3:** Correlation between mean scores of WQOLI and SF36

		WQOL	SF36
WQOLI	Pearson Correlation	1	.717^a^
	Sig. (2-tailed)		.000
	N	590	590
SF-36	Pearson Correlation	.717^a^	1
	Sig. (2-tailed)	.000	
	N	590	590

^a^ Correlation is significant at the 0.01 level (2-tailed)

**Table 4:** Determine the internal consistency: Cronbach’s alpha of “WQOLI”

Sub-scale	Item number	Cronbach’s alpha, *N* = 30
First factor: sense of peace in life	23	α = 0/882
second factor: sense of security	21	α = 0.839
third factor: health responsibility	15	α = 0.859
forth factor: pleasant communication	18	α = 0.893
fifth factor: received comprehensive support	13	α = 0.843
Total	90	α = 0.919

**Table 5:** Determination of stability (test-re-test) of the entire instrument with a two-week interval

		pre	post
pre	Pearson Correlation	1	.889^a^
	Sig. (2-tailed)		.000
	N	30	30
post	Pearson Correlation	.889^a^	1
	Sig. (2-tailed)	.000	
	N	30	30

^a^ Correlation is significant at the 0.01 level (2-tailed)

### Responsiveness

In this research, out of 649 women, 59 gave incomplete answers to the questionnaires, so the response rate was 91%. The average time to answer the questionnaire was estimated about 45 min. Also, the questionnaire is designed in such a way that another person can read the items for the main sample and mark the answers. This questionnaire is designed according to the culture of Iranian women and it is not invasive. In general, the designed questionnaire is appropriate in terms of responsiveness.

### How to calculate the measurement score of women’s quality of life

The measurement scale in the present questionnaire was considered a Likert scale with a five-part spectrum of negative to positive with the midpoint. According to the experts' opinion, when evaluating the content of the tool, the items were formulated as positive, so the score for the items was done only positively. Scores are considered: I quite agree (5), I agree (4), I neither agree nor disagree (3), I disagree (2), I quite disagree (1). The total score is determined by calculating the average total score of all items. The minimum score of the questionnaire is 90 and the maximum is 450. If the score is between (90 and 162), the QOL will be very unfavorable. If the score is between (162.1 and 234), QOL will fit in the relatively unfavorable spectrum. Gaining a score of between (234.1 and 306) points, QOL is in the middle range. If the score is between (306.1 and 378), it indicates the desirability of a person's QOL. If the individual score is (378.1 and 450), the QOL is very favorable.

## Discussion

Despite the importance of women's health and quality of life and their role as the base of family health, a specific definition of the concept of women's quality of life has not yet been provided. Therefore, it is not possible to compare with the definition mentioned in the current research. However, several researchers have tried to define the overall quality of life in their research. In the present study, from the women's point of view, five important dimensions of quality of life included: a sense of peace in life, a sense of security, comprehensive support, responsibility for health, and pleasant communication. France, citing Kimura has proposed four dimensions for the concept of QOL: "health and performance", "socio-economic", "psychological", and "family dimension" [29]. Also, Padilla quoted by Laudaniti has paid attention to five dimensions including psychological well-being, social concerns, coping with appearance, physical well-being, and response to treatment in defining the quality of life [30]. As is evident in the above studies, areas such as physical and psychological health, personal relationships, social support, and security are mentioned, but the quality of life of women has not been studied with a deep view from the gender angle and what concerns women. Therefore, the difference between this qualitative study and others is due to a specific look at the quality of life of women in Iran.

In the qualitative research by Rowshani et al., women stated the health and success of their children, meeting the needs of all family members, contentment and gratitude, and patiently bearing difficulties are components of a sense of peace in their lives [31] and in a research Niazi and Farshadfar stated that there is a significant relationship between the level of social trust and the feeling of social security among women in the northern and southern regions of Tehran [32]. The result of a study conducted by Jadidi & Ameri showed that the level of social support from the family is related to understanding the meaning of life among ill women [33] which is in line with the data of the present study.

The results of Pekel's study indicated that teachers with higher life satisfaction were more likely to engage in health-promoting behaviors [34]. Therefore, there is a significant positive relationship between responsibility and life satisfaction. In the present study, women also mentioned that attention to health needs is one of the components of having a good quality of life. Pekel also showed in his study that interpersonal relationships have a significant relationship with people's life satisfaction [34]. He states that whatever situation a person is in, proper communication makes his life easier, more enjoyable, and more successful. In the present study, women considered having free communication with the people around them, with God, and receiving energy from their environment and free use of mass communication tools necessary to have a sense of happiness in life.

As the review of studies shows, all of the above researches have studied the variables affecting satisfaction and quality of life separately, qualitatively, or quantitatively, but none of them have comprehensively defined the quality of life from the women's point of view, and no specific tool has been designed based on it. However, from a content perspective, we have a variety of quality-of-life tools. In a general classification, they can be divided into 3 categories. A-Tools that are general and measure several aspects of quality of life like WHOQOL-BREF or SF_36_ [9, 10]. B-Tools that are specific to a particular age group and usually measure a specific dimension, for example, KIDSCREEN-52, specific for the age group of 8 to 18 years [35], the Pediatric Quality of Life Inventory (PedsQL) Family Impact Scale [36] and QLI-YES which is specific for assessing the quality of life of young elderly in Sri Lanka [25]. C- Tools that relate to a particular group of patients. To mention as an instance, the specific quality of life measure for Stroke [37], and the fertility quality of life (FertiQoL) tool [12]. Although each of these tools is appropriate and valuable for their particulars, they do not adequately assess the quality of life of women as one of the most vulnerable groups in society with their specific features. The “WQOLI” is a new measure of perceived QOL amongst women because items of the questionnaire were created based on the result of qualitative content analysis.

For a better comparison among the three categories of quality-of-life tools mentioned, the general tools of the World Health Organization Quality of Life Questionnaire Short Form (WHOQOL-BREF) and the Short Form Quality of Life Questionnaire (SF_36_) which are more used in healthy and sick communities are criticized.

The short form of the World Health Organization Quality of Life Questionnaire (WHOQOL-BREF) is an example of a general tool for measuring the quality of life, which was created in 1996 by a group of experts of the World Health Organization from 15 international centers under the supervision of Dr. Orly and by adjusting the items of the 100-question form [38, 39].

In terms of content, the WHOQOL-BREF contains 26 questions and examines the quality of life in four areas related to health, "Physical health", "Mental health", "Social relations", and "Living environment". The first question is about the quality of life in general and the second question is about the health status in general. The next 24 questions evaluate the quality of life in the four areas mentioned. The physical domain includes seven questions related to daily life activities, dependence on drugs and medical aids, energy and exhaustion, dynamics, pain and discomfort, sleep and rest, and work capacity. The psychological field includes six questions related to body image and appearance, negative emotions, positive emotions, self-esteem, spirituality, religion, personal beliefs, and thinking and concentration. The field of social relations includes three questions related to personal relations, social support, and sexual activity.

The field of living environment includes eight questions related to financial resources, freedom, physical safety and security, accessibility, and quality of health and social care, home environment, the opportunity to learn new skills and information, the amount of participation, and the opportunity for recreational and entertaining activities, the environment physical (pollution, noise, traffic, weather) and transportation [38, 39].

Although this questionnaire covers a wide range of aspects of quality of life, the number of items in each area, especially the area of social relations, is very small and has only three items, but it has also paid attention to the area of the living environment. In the WHOQOL-BREF, social relations and living environment are mentioned separately, but in the WQOLI, the dimension of pleasant communication covers all social relations and even the relationships that a person establishes with his physical environment.

In the questionnaire of the World Health Organization, two special dimensions of physical health and mental health are mentioned separately. Also, the spiritual aspect of health and quality of life has not been considered. An item entitled "How satisfied are you with your ability to perform daily activities?" is located in the field of the living environment, which seems to be better if it is mentioned in the physical or psychological field. In the WQOLI, the feeling of peace in life is mentioned, which covers almost all aspects of physical, mental, and spiritual health.

In terms of the credibility of the WHOQOL-BREF has been measured in a sample of experts from 23 countries and the reliability of more than 0.70 and its validity have been confirmed [38, 39].

For the first time, this questionnaire was translated by Nejat (2006) and was psychometrically evaluated by examining 1167 people from Iranian society. The reliability of the mentioned questionnaire was calculated using Cronbach's alpha in the fields of physical health, mental health, and living environment, and above 0.7, but in the field of social relations, it was 0.55 [38, 39]. In this questionnaire, issues such as face validity, primary reliability, agreement between evaluators, and responsiveness have not been addressed. On the other hand, the whole tool was not built based on the real experiences of women, and its items were compiled only by using the opinions of a group of experts from the WHO committee. As mentioned, quality of life is a subjective concept that can be understood differently in different groups of society. The WQOLI has been measured quantitatively and qualitatively with the live experience of women according to the cultural and religious conditions of Iranian society [8]. On the other hand, the reliability of the tool has been measured in both preliminary and final ways with valid methods such as internal consistency and intra-cluster correlation and has obtained an alpha of more than 0.7.

Short Form Questionnaire of Quality of Life (SF_36_) is the second tool that is compared with the WQOLI. This tool is another category of general tools that was designed in 1992 in the United States by Ware & Sherbourne to measure the health-related quality of life of healthy and sick people. This questionnaire is one of the standard and general tools for measuring the quality of life and contains 36 questions, which have eight dimensions of general health (2 items), feeling of vitality (8 items), physical performance (10 items), role limitation due to physical problems (4 items), physical pain (2 items), mental health (5 items), role limitation due to mental problems (3 items) and social functioning (2 items) [40]. Currently, this tool is the most widely used tool for measuring health-related quality of life in the world and also in our society. In this form, none of the items have been mentioned as a person's satisfaction with their health status and other factors related to the quality of life, in other words, it has only covered the aspects related to health, and on the other hand, the objective health status of the person is more included than the subjective understanding of the quality of life.

In the WQOLI, these concepts are more comprehensively placed in the dimension of responsibility towards health, which from the women's point of view has been effective in the quality of life. However, factors such as comprehensive support, a sense of security, and pleasant communication are not mentioned in the short form of quality of life (SF_36_).

In Montazeri et al.'s study, the area of vitality, which is present in the short form SF_36_, is a vague concept, and the reason for that is the translation of the concept from English to Persian [40]. In the present study, things like happiness and vitality are included in the sense of peace in life, but because it is based on the real experiences of the participants in the present study, it has resolved the ambiguities that existed in the SF_36_ tool due to the translation of a non-native tool. Although this questionnaire has also been used in many studies, some of its dimensions such as the mental dimension or the social performance dimension only contain three questions, which seems to be not comprehensive enough in examining the above areas. On the other hand, this tool does not fully cover the different aspects of the quality of life, especially the dimension of spiritual communication, which can be important in the cultural and religious conditions of our Muslim society. Also, it has not paid attention to the aspect of communication with the environment that can have a special effect on the quality of life, most importantly, this tool is not designed qualitatively and based on the subjective experiences of the participants, and therefore it is not suitable for the Iranian women's society. The short form SF_36_ was translated into Persian language by Montazeri et al. in 2005 and its validity and reliability were investigated. Cronbach's alpha reliability of all the dimensions except the field of vitality is more than 0.7, which the researcher attributed to the difficulty in translating the word vitality from English to Persian [40]. On the other hand, this questionnaire, like the WHOQOL-BREF, did not mention face validity, basic reliability, agreement between evaluators, and factor analysis.

All in all, WQOLI is a more comprehensive tool than the ones mentioned above because it covers all aspects of the quality of life. WQOLI incorporates a sense of security, and health responsibility, and received comprehensive support that is not mentioned in the WHOQOL-BREF and SF_36_. These three aspects of the women's perspective have a special significance in their quality of life and even affect other aspects of their life and health.

### Limitations

In this study, the psychometric analysis targeted women in a central city in Iran. Consequently, this study may not be generalizable to other women living in rural and urban areas. Also, his questionnaire focuses on domains that come from the qualitative descriptions of a wide age range of women but we are not considered pregnant women. Therefore, the use of this questionnaire in pregnant women should also be tested. The study population only included Muslim women so it is suggested to study the quality of life of women with other religions. In addition, some of our samples may have chronic diseases that they have hidden from the researcher. Therefore, more research is needed to increase the efficiency of this questionnaire in healthy and sick populations. Also, this study was conducted before the outbreak of the 2019 coronavirus disease. Considering the fear and anxiety caused by the disease epidemic, it is recommended to use this questionnaire to evaluate its applicability in such epidemics.

## Conclusions

In conclusion, herein we have demonstrated the designing of a scale that is specific for assessing women’s QOL, named the WQOLI, which comprises 90 items, the responses to which are given on a 5-point Likert-type scale. The underlying structure of the WQOLI scale comprises five domains: “sense of peace in life, sense of security, health responsibility, pleasant communication, received comprehensive support". In this study, 590 out of 649 women answered the questionnaire completely. So, this mirrors a high degree of acceptance and simplicity that respondents can complete it. The average response time to the questionnaire was 45 min. Therefore, it is suggested to prepare a shorter form of this questionnaire to decrease the answering time. We also offer suggestions about the administration of this instrument by new types, such as a computerized scale instead of the usual paper-and-pencil type. The questionnaire is also designed in such a way that another person can read the items for the original sample and mark the answers. On the other hand, since the questionnaire is tailored to the women's culture in this study and does not invade them, the designed questionnaire is relatively feasible for the general women population.

### Implications of this study

From the theoretical aspect, this study provides a definition of the quality of life of Iranian women that has not been addressed so far, which can be a basis for designing models to improve the quality of life and women's health.

From a practical point of view, this scale (IWQOLI) can be used as an objective tool to evaluate women's quality of life and investigate potential health problems by community health and family health nurses at the primary prevention level.

## Data Availability

The set of data generated during the current study will not be available to the public due to ethical issues and adherence to the principle of confidentiality, but it can be provided upon reasonable request from the corresponding author.

## Abbreviations

IWQOLI: Iranian Women’s Quality of Life Instrument
QOL: Quality of Life
